# Characterizing street-connected children and youths’ social and health inequities in Kenya: a qualitative study

**DOI:** 10.1186/s12939-020-01255-8

**Published:** 2020-08-28

**Authors:** L. Embleton, P. Shah, A. Gayapersad, R. Kiptui, D. Ayuku, P. Braitstein

**Affiliations:** 1 Division of Epidemiology, Dalla Lana School of Public Health, University of Toronto, Health Sciences Building, 155 College Street, 5th Floor, Toronto, ON M5T 3M7 Canada; 2 Institute of Medical Sciences, Faculty of Medicine, University of Toronto, 1 Kings College Circle Room 2374, Toronto, ON M5S 1A8 Canada; 3 London School of Hygiene & Tropical Medicine, Keppel St, Bloomsbury, London, WC1E 7HT UK; 4 Academic Model Providing Access to Healthcare (AMPATH), P.O. Box 4606-30100, Eldoret, Kenya; 5 Department of Behavioural Science, School of Medicine, Moi University, College of Health Sciences, P.O. Box 4606-30100, Eldoret, Kenya; 6 Department of Medicine, School of Medicine, Moi University, College of Health Sciences, P.O. Box 4606-30100, Eldoret, Kenya

**Keywords:** Street children, Kenya, Social determinants of health, Health equity, Human rights

## Abstract

**Background:**

Street-connected children and youth (SCY) in Kenya disproportionately experience preventable morbidities and premature mortality. We theorize these health inequities are socially produced and result from systemic discrimination and a lack of human rights attainment. Therefore, we sought to identify and understand how SCY’s social and health inequities in Kenya are produced, maintained, and shaped by structural and social determinants of health using the WHO conceptual framework on social determinants of health (SDH) and the Convention on the Rights of the Child (CRC) General Comment no. 17.

**Methods:**

This qualitative study was conducted from May 2017 to September 2018 using multiple methods including focus group discussions, in-depth interviews, archival review of newspaper articles, and analysis of a government policy document. We purposively sampled 100 participants including community leaders, government officials, vendors, police officers, general community residents, parents of SCY, and stakeholders in 5 counties across Kenya to participate in focus group discussions and in-depth interviews. We conducted a thematic analysis situated in the conceptual framework on SDH and the CRC.

**Results:**

Our findings indicate that SCY’s social and health disparities arise as a result of structural and social determinants stemming from a socioeconomic and political environment that produces systemic discrimination, breaches human rights, and influences their unequal socioeconomic position in society. These social determinants influence SCY’s intermediary determinants of health resulting in a lack of basic material needs, being precariously housed or homeless, engaging in substance use and misuse, and experiencing several psychosocial stressors, all of which shape health outcomes and equity for this population.

**Conclusions:**

SCY in Kenya experience social and health inequities that are avoidable and unjust. These social and health disparities arise as a result of structural and social determinants of health inequities stemming from the socioeconomic and political context in Kenya that produces systemic discrimination and influences SCYs’ unequal socioeconomic position in society. Remedial action to reverse human rights contraventions and to advance health equity through action on SDH for SCY in Kenya is urgently needed.

## Background

Children (persons ≤18 years of age) and youth (persons between the ages of 15 and 24) living and working on the streets have been known by various terms and definitions, which have been used to convey their circumstances and connections to the streets and public spaces. Terminology has evolved to reduce stigmatization and negative connotations associated with the label ‘street child’, and the terms ‘street-connected’ or ‘children and youth in street situations’ have been adopted to identify children and youth for whom the streets plays a significant role in their everyday lives and social identities [1, 2]. Street-connected children and youth (SCY) in low- and middle-income countries (LMICs), are a socially and economically distinct group of young people, who experience numerous health inequities that are avoidable [2–4]. SCY in LMICs report that structural and social inequities, namely abject poverty, family conflict, and abuse, precipitate their migration to the streets [5]. Subsequently, the context in which children and youth find themselves living and working on the streets exacerbates the social, economic, and health inequities experienced by this population [3, 6–8].

SCY are prevalent in Kenyan cities [9–11], however no accurate national estimate of the number of children and youth connected to the streets has been published. Known by the public as *chokoraa* (garbage pickers), these children and youth are subject to human rights violations and experience tremendous stigmatization, social exclusion and discrimination, all of which have an impact on their health and well-being [6, 10, 12–14]. This marginalized group disproportionately experiences preventable morbidities including but not limited to: a heightened prevalence of human immunodeficiency virus (HIV) and sexually transmitted infections, post-traumatic stress disorder, substance use and misuse, and negative sexual and reproductive health outcomes [9, 15–23]. Moreover, SCY in Kenya succumb to death prematurely through preventable causes of mortality [24, 25]. SCY also experience social and economic marginalization and participate in a street-based or informal labor economy, where they earn on average between 50 and 100 Kenyan shillings (Ksh) per day (~ US$0.50 – US$1.00) [6, 26]. SCY are frequently involved in the criminal-justice system and report experiencing conflict with the police, arrest and incarceration, and harassment, violence, and beatings from authorities [6, 13, 14, 22, 27, 28]. Despite the robust evidence of social and health inequities experienced by SCY, to our knowledge no studies have been conducted to date that explore how the social and health inequities experienced by SCY in Kenya are produced, maintained, and shaped by structural and social determinants of health (SDH) in this context. We theorize that the extensive social and health inequities experienced by SCY in Kenya are socially produced and result from systemic discrimination and a lack of human rights attainment for this marginalized population.

The WHO conceptual framework on SDH can be used to explore and identify how the social, economic, and political context in a specific country influences socioeconomic positions in society, whereby populations are stratified by social class, gender and sexual identity, ethnicity (racism), income, education, and occupation [29]. Contextual factors and structural mechanisms that give rise to social stratification (e.g. social class, income, education, etc.) and an individual’s socioeconomic position are the structural and social determinants of health inequities. An individual’s socioeconomic position, in turn shapes specific determinants of health status known as ‘*intermediary determinants of health*’. These intermediary determinants include material and psychosocial circumstances, such as housing, food availability, stressful living circumstances, and social support, and behavioral and biological factors, such as nutrition and drug and alcohol consumption. As a result of SDH and intermediary determinants of health, individuals experience differences in exposure and vulnerability to health-compromising conditions, which ultimately impact health equity [29].

The WHO describes health inequities as “health differences that are socially produced, systematic in their distribution across the population, and unfair” [29]. When individuals in a society have unequal rights and access to key determinants of health, including but not limited to freedom from discrimination, food, clothing, housing, education, and medical care, health inequities arise. Moreover, an individuals’ right to enjoy the highest attainable standard of physical and mental health, is influenced by the socioeconomic, political, and environmental conditions in a particular context [29]. Given the strong link between achieving health equity and attainment of human rights, a human rights framework can be used with the conceptual framework on SDH to explore and analyze the underlying processes of systemic discrimination and the social production of health inequities in a specific socioeconomic-political context [29].

The Convention on the Rights of the Child (CRC), stemming from the Universal Declaration of Human Rights, recognized that children are in need of special protection and assistance, and came into force in 1990 [30]. Kenya is a signatory of the CRC [31], and all children under the age of 18 years should be safeguarded as per the CRC under the Kenya Children’s Act, which legally outlines children’s rights and welfare in the country [32]. In 2017, a General Comment on Children and Street Situations was released by the Committee on the Rights of the Child to provide authoritative guidance to States to respond to injustices experienced by SCY and improve their circumstances using a child rights approach building on the CRC [2]. The General Comment no. 21 recognizes SCY’s rights to several structural, social, and intermediary determinants of health, such as non-discrimination (Article 2), the right to education (Article 28), and the right to an adequate standard of living (Article 27) [2].

Given the social and health inequities experienced by SCY in Kenya and a lack of research conducted to understand how these inequities arise in this context, we sought to explore and understand how health inequities experienced by SCY in Kenya are produced, maintained, and shaped using the WHO conceptual framework on SDH and the CRC General Comment No. 21 (2017) on Children in Street Situations [2, 29].

## Methods

### Study setting

The study was conducted across five counties in Kenya: Trans-Nzoia, Bungoma, Kisumu, Uasin Gishu, and Nakuru. We interviewed participants in the respective capital of each county: Kitale, Bungoma, Kisumu, Eldoret, and Nakuru. These study sites were purposively selected given the large numbers of SCY known to live and work in these towns in western Kenya [9, 10]. The counties’ respective populations and poverty demographics are shown in Table 1. Eldoret, the capital of Uasin Gishu, was the primary study site. It is home to Moi University, Moi Teaching and Referral Hospital (MTRH), and the Academic Model Providing Access to Healthcare (AMPATH), a long-standing partnership between Moi University, MTRH, and a consortium of universities from North America [33].

**Table 1:** Population and percentage of persons living in poverty for five study sites

	Bungoma	Kisumu	Trans-Nzoia	Uasin Gishu	Nakuru
Population	1,375,064	968,909	818,757	894,179	1,603,325
% Poverty	52.2	45	50.1	44.6	41.8

### Epistemological principles and study design

The study rests on critical theoretically engaged qualitative research principles [34]. Given our interest in underlying power structures and how they impact SCY’s social and health inequities, the study privileged qualitative methodology to explore and describe the public perceptions of, and proposed and existing responses to, the phenomenon of SCY in Kenya. We opted for an explanatory and descriptive qualitative design using multiple data generation methods to illuminate how the social and health inequities experienced by SCY in Kenya are produced, shaped, and maintained. Qualitative research is particularly well suited to explore and produce knowledge on how power structures in this context contribute to and produce inequities on this issue [34, 35].

### Sampling considerations and study participants

This study sought to purposively sample a diverse range of participants who had knowledge of and experience interacting with SCY. The overall aim in using purposive rather than probability sampling was to include information-rich cases for in-depth study [36].

The research team has a long-standing relationship with the street community in Eldoret, Kenya where they have conducted participatory research with this population for over 15 years. Our established relationship with the local community in Uasin Gishu County enabled us to reach a diverse group of participants. In Uasin Gishu County we included community leaders (Chiefs and Elders), County Children’s Coordinator, Children’s Officers, police officers, vendors, general community members, stakeholders, parents of street children, former and current SCY, peer navigators, and healthcare providers at MTRH and AMPATH. Across the other counties we engaged Children’s Officers, police officers, and SCY. SCY were eligible to participate if they were aged 15 to 24, and other social actors had to be aged at least 18.

### Recruitment and enrolment

We purposively recruited community members, including, community leaders, government officials, vendors, police officers, general community residents, parents of SCY, and stakeholders, and contacted them by phone or in person to explain the purpose of this study and to invite them to voluntarily participate. We contacted government officials initially with a formal letter informing them of the purpose of the study and followed up in person. Healthcare providers and peer navigators working at MTRH and AMPATH were recruited through our established networks and contacts. We invited social workers, clinical officers, nurses, and HIV testing and counselling practitioners in order to gather a broad range of opinions from individuals providing healthcare. Government officials in Uasin Gishu were consented and interviewed in their offices, while all other participants in Uasin Gishu were invited to the referral hospital, or Moi University offices for consent and interviews. Participants in other counties were consented and interviewed in their offices and places of employment.

SCY aged 15–24 in all counties were purposively sampled from street venues called “bases/barracks” (primary locations in which street children reside). Street outreach and study sensitization occurred at these sites to establish rapport and trust with SCY. In these street venues, the purpose of the study was explained, and SCY were invited to participate voluntarily in the investigation. In Uasin Gishu, SCY were invited to the Rafiki Centre for Excellence in Adolescent Health at MTRH to undergo enrolment, consent, and participate in interviews. In other counties, SCY were enrolled, gave consent, and provided interviews in street venues.

The number and breakdown of participants, type of interview, and their location is presented in Table 2. In total, the study recruited 100 participants, 48 women and 52 men. The median age of community members interviewed was 42 years, and SCY 16 years. The number of participants selected for this study was adequate to permit deep case-oriented analysis and to produce credible and analytically significant findings resulting in a new and richly textured understanding of how social and health inequities experienced by SCY in Kenya are produced, shaped, and maintained [37].

**Table 2:** Breakdown of participants, interviews, and location

Social Actors	# of interviews	Location	Gender of Interviewees
Community leaders	4	Uasin Gishu	4 Men
County Children’s Coordinators	1	Uasin Gishu	1 Man
Police Officers	6	Uasin Gishu, Nakuru Trans-Nzoia, Kisumu, Bungoma	3 Women, 3 Men
Children’s Officer(s)	6	Uasin Gishu, Nakuru Trans-Nzoia, Kisumu, Bungoma	2 Women, 4 Men
Vendors	2	Uasin Gishu	1 Woman, 1 Man
General Community	3	Uasin Gishu	1 Woman, 2 Men
CBO / Stakeholders & SCY Advocates	6	Uasin Gishu	2 Women, 4 Men
Peer Navigators	2	Uasin Gishu	1 Woman, 1 Man
Parents of Street children	1	Uasin Gishu	1 Woman
Former Street-connected youth	3	Uasin Gishu	2 Women, 1 Man
Street-connected youth	7	Uasin Gishu, Kisumu, Trans-Nzoia	5 Women, 2 Men
**Total In-depth Interviews**	**41**		**18 women, 23 men**
AMPATH Clinicians	FGD	Uasin Gishu	2 women, 3 men
AMPATH Nurses, Social Work, Counsellors	FGD	Uasin Gishu	4 Women, 2 Men
MTRH Clinicians	FGD	Uasin Gishu	6 Men
MTRH Nurses	FGD	Uasin Gishu	6 Women
SCY Males FGD	FGD	Uasin Gishu	12 men
SCY Females FGD	FGD	Uasin Gishu	12 women
Mixed gender SCY Nakuru	FGD	Nakuru	6 Young women, 6 Young men
**Total Number of FGDs**	7		**30 women, 29 men**

### Ethical considerations

This study received ethics approval from Moi University-MTRH Institutional Research Ethics Committee and University of Toronto Research Ethics Board. The study received a waiver of parental consent for minors. SCY participants were asked to provide documented verbal consent (those aged 18 to 24) or assent (those aged 15 to 17) at each encounter. Research on cognition and capacity suggests adolescents and younger children show significant ability to provide informed consent [38]. We have established a process for conducting ethical research with SCY including a process for informed consent/assent [39]. Qualified team members were present at all interviews with SCY to make qualitative determination as to whether the youth understood what they were assenting to. Written informed consent was obtained from all other participants. Participants were made aware that their interviews would be audio-recorded; nine participants (police officers and children’s officers) declined to be audio-recorded but agreed to be interviewed and gave the interviewer permission to take notes. Community participants and SCY were compensated for their time with 200 Ksh (~US$2.00) and government officials 1000 Ksh (~ $US10.00).

### Data generation

From May 2017 to September 2018 we used multiple data generation methods, including focus group discussions, in-depth interviews, archival review of newspaper articles, and analysis of a government policy document to generate a data corpus for this qualitative analysis.

The study team, trained experts in qualitative research methodology, conducted focus group discussions and in-depth interviews in either English or Swahili. We conducted 41 in-depth interviews and seven FGDs (Table 2). In total, 22 interviews were conducted in English and 26 were conducted in either Swahili or a mix of Swahili and English. Focus group discussions took on average one and a half hours and in-depth interviews 40 min. Focus group discussions and in-depth interviews used an interview guide that asked participants about their general perceptions of the population, their experiences interacting with SCY, and their perceptions of their needs. For healthcare providers, additional questions were included in relation to the provision of healthcare. A separate interview guide was developed for SCY, which asked about their experiences and interactions with the community, their perceived needs, and their ability to access healthcare and other social services. The interview guides are available in Additional File 1.

In addition to focus group discussions and in-depth interviews, we included 11 newspaper articles published from 2015 to present and a government policy document [40–50]. Kenya lacks a national policy on SCY [51]. The Street Families Rehabilitation Trust Fund (SFRTF) was established in 2003, which sought to address the needs of SCY and street families and safeguard their rights, however no official policy documents exist with respect to this program [51]. The existing and strong relationship the research team has in Uasin Gishu County with stakeholders and government officials made the research team aware of a local Uasin Gishu county policy document that was included in the present analysis. The government policy document was provided to the research team through the Uasin Gishu Children’s Forum. To the best of our knowledge, there are no other policy documents available in the other counties, however it is possible they exist and are not publicly available.

Given that media influence public perceptions, exploring news articles on SCY can shed light on how they are portrayed, policies that have been implemented, and interactions that SCY have with the community at large in this context [52]. Newspaper articles provided additional information on the sociocultural and political context in Kenya that influenced the social and health inequities experienced by SCY. These newspaper articles were randomly selected from a set of national newspaper publications that covered issues related to the portrayal, the living conditions and treatment of SCY in Kenya, with the exception of one international article, which was purposively included. The use of simple random sampling strategy provided a representative of articles on SCY. Due to limited time and resources it was not possible to review and analyse all newspaper articles related to SCY.

### Research team and reflexivity

The research team are committed to improving the health and well-being of SCY in this region and other LMICs. The multi-disciplinary research team who conducted this research consists of 4 women and 2 men from Kenya and Canada, whom have expertise in public and population health, child and adult psychology, social and behavioral sciences, epidemiology, and human rights. We understood that our positionality may have impacted the interview, that participants’ responses may have been mediated by our presence. To empower SCY we conducted focus groups and interviews in familiar environments or one of their own choosing. As one-on-one interviews can be intimidating especially for young people, we conducted focus group discussions with SCY to offset the power imbalance that may exist between the research team conducting the interviews and our participants [52]. Constructive rapport was established through the relationship built over several years of participatory action research with SCY in this setting creating a bond of trust. This encouraged participants’ disclosure, to tell personal and detailed stories. Participants were assured that their response would be anonymous and that, if at any time during the interview they were uncomfortable and no longer wished to participate, they could leave without any repercussions.

### Qualitative data analysis

Transcription and translation are deeply interpretative processes [53, 54]. Interviews and focus group discussion were transcribed verbatim. All audio files and transcripts were reviewed by the authors to ensure quality. RK transcribed interviews and focus group discussion completed in English. RK (Kenyan) also translated and transcribed those completed in Swahili. Quality check performed by LE (intermediate Swahili language skills) and PS (Kenyan). Several years of conducting participatory research with this population enabled the authors to contextualise and interpret the data. Iterative processes and continuous questioning of the understanding of data and reviewing of findings, provided opportunities for minimising descriptive and interpretive biases.

A deductive qualitative data analysis approach was conducted. In analytic meetings, using the CRC General Comment No. 21 on Children in Street Situations (henceforth referred to only as CRC), we mapped specific human rights articles onto the WHO conceptual framework for SDH to guide our analysis to understand the processes of systemic discrimination and production of health inequities.

Interview transcripts and newspaper articles were read multiple times by the research team to achieve immersion prior to code development. The deductive approach to identify the coding scheme allowed for the development of codes corresponding to specific concepts which were then used to generate themes [55]. We developed a series of codes using specific articles outlined in the CRC to capture upholding or contravening SCY’s human rights. To capture SDH, we coded broadly for context to include all social-cultural, economic, and political mechanisms that maintain social order with the following context sub-codes: criminal-legal, cultural, economic, political, and religious. We developed the final codebook by repeatedly testing its validity and comprehensiveness through test-coding transcripts. Transcripts, newspaper articles and the policy document were coded by four of the authors (LE, AG, RK, PS) and compared for consistency. Analytic notes and annotations made during coding by each author were used in a series of interpretive meetings to define and refine themes. To enhance the reliability of the data, we triangulated data from multiple sources (e.g. interviews with different types of participants) as a data validation strategy [36]. We also triangulated data from multiple data collection methods. By combining focus groups with individual interviews we maximized the strengths of each while overcoming their unique deficiencies [56]. To increase validity and reliability of the analysis, inter-rater reliability was also employed, a type of researcher triangulation by which multiple researchers are involved in the analytical process [57].

#### Findings

**Fig. 1:**
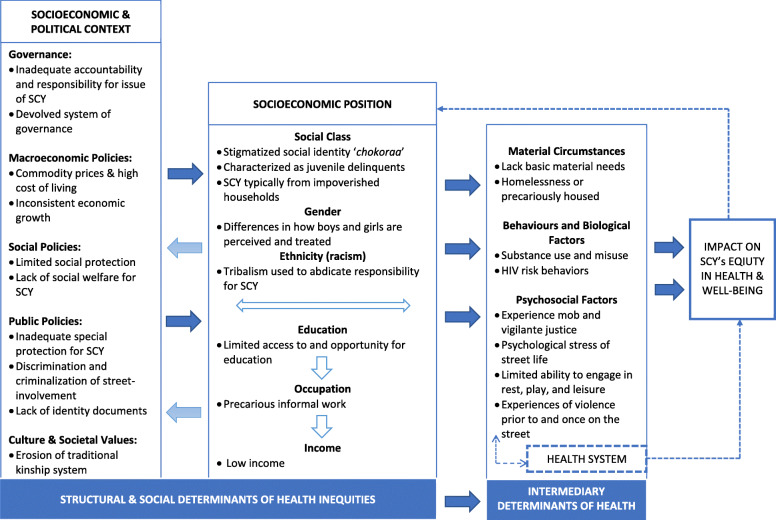
Summarizes the study’s findings in the WHO conceptual framework on structural and social determinants of health

Summarized in the SDH framework (Fig. 1), our analysis is divided into 3 major themes 1) socioeconomic and political context; 2) socioeconomic position, 3) and intermediary determinants of health to characterize how the structural and social determinants of health inequities and intermediary determinants of health impact SCYs equity in health and well-being in this context. In the first theme exploring the structural determinants of health inequities, we characterized the influence and role of the socioeconomic and political context in Kenya in producing and maintaining structural determinants of health inequities through the four sub-themes: governance; macroeconomic policies; social policies, societal and cultural values; and public policy. Our analysis uncovered the exertion of power over SCY as evidenced by human rights contraventions. In the major theme of ‘socioeconomic position’ we identified multiple and intersecting forms of discrimination and how they shape and influence SCY’s position in society in two sub-themes: social class, gender, and ethnicity; and education, occupation, and income. Table 3 summarizes our findings and shows how structural and social determinants and corresponding human rights contraventions impact SCY’s social and health inequities. Lastly, we explored the intermediary determinants of health through two sub-themes of material and psychosocial circumstances. Table 4 summarizes our findings with respect to the intermediary determinants of health, human rights contraventions, and impact on SCY’s social and health inequities.

**Table 3:** Structural and social determinants of health inequities, human rights, and their impact on SCY’s social and health inequities

Structural Determinants of Health Inequities			
Domain	Human Rights	Supporting Quotes	Impact on social and health inequities
**Governance**	***Article 4 on appropriate measures*:** state parties shall undertake all appropriate legislative, administrative, and other measures for the implementation of the rights recognized in the Convention.	“The government has no interest; they are being looked at as a problem. The government handles these issues with backwardness, they want to tackle them on the streets and push them home instead of solving things that are attracting them on the streets and creating more systems to prevent them from coming to the streets.” (Stakeholder 2)	• Political inaction and poor public policies carried out by the state impact SCY’s life circumstances, socioeconomic position, and therefore social and health inequities. • SCY and their health is not a priority in the government’s agenda, with limited resources allocated to their issues. • Lack of political will and a disregard of responsibility of the State for the phenomenon of SCY, despite legal obligation as a CRC signatory.
**Social Policy**	***Article 18 on parental responsibility:*** states are obliged to provide assistance to parents and guardians to prevent children ending up in street situations.	“At the national level we have what we call The Street Family Trust Fund, which is based in Nairobi. It’s supposed to come to the major cities and work together with us so that we can have such programs.” (County Children’s Officer)	• Inadequate and unimplemented social welfare programs for SCY and their families leaves them without a social safety net. • On-going structural forces place pressure on households leaving them unable to adequately provide and care for children’s needs.
**Public Policy**	**Art*icle 7 on birth registration and 8 on identity:*** states should ensure free, accessible, simple and expeditious birth registration is available to all children at all ages and street-connected children and youth should be supported to obtain legal identity documents.	“Some have reached the age of getting IDs, for one to get an ID one has to have a birth certificate and the parent’s IDs so most of these children can’t get them. Also, when they are sick, they don’t get treatment easily so the government should work on that.” (Community Leader 3)	• SCY lacking identity have difficulties accessing education, health, other social services, justice and family reunification all of which have a long-term impact on their socioeconomic position and health and well-being.
**Public Policy**	***Article 15 on the right to freedom of association and peaceful assembly:*** states should ensure that street-connected children and youths’ access to public space in which to associate is not denied in a discriminatory way.	“So, like, the county government, what it did, it was, I’ll use that term ‘making it unfriendly for them in town’ so that once you see them even police officers, enforcement officers, they are put in strategic places. So, these children, totally they will not step into the central business district (CBD).” (Children’s Officer)	• Limiting SCY’s access to public spaces and use of police and other officers to enforce this restriction is discriminatory and contravenes to their right to associate in public places. • Practices that limit SCY’s access impacts their social, psychological, and physical health if they are unable to associate freely in their social networks or access particular services within restricted public spaces.
**Public Policy**	***Article 20 on the right to special protection and assistance for children deprived of a family environment (44.) types of care:*** states are the de facto caregiver and are obliged to ensure alternative care to a child temporarily or permanently deprived of his or her family environment. Deprivation of liberty, in detention cells or closed centres, is never a form of protection.	“Ideally, we have programs and activities that we can do, the only challenge we have is resources. The biggest challenge here is if you have to rescue them, you have to take them to a safe place, and you have to find time to engage them and find out why they are on the streets. As we speak now, we don’t have a holding facility. The rescue center is not in a position to hold all street children. The rescue center is not just for street children bit for any child that requires to be rescued because children are also abused in their families.” (County Children’s Officer)	• Inadequate shelters, rescue centers, and alternative care environments leave SCY without protection, shelter, and other basic needs thereby impacting their health and well-being. • The use of prison, remand homes/juvenile detention, or cells, are inappropriate alternative care environments and impacts SCY’s social, psychological and physical health and well-being.
**Public Policy**	***Article 20 on the right to special protection and assistance for children deprived of a family environment (45.) applying a child rights approach:*** states should ensure that children are not forced to depend on the street for survival and that they are not forced to accept placements against their will. States should ensure that State and civil society-run shelters and facilities are safe and of good quality.	There is a case I have witnessed, he came to the streets, but the family is well off, he had no valid reason but just said he liked the streets more than his home. We took him home twice but still went back to the streets. I don’t pity him because he has parents and a home, he claimed it was due to hostility by the parents, but they denied that (Clinical Officers)	• Unsafe, inappropriate and poor-quality shelters and facilities for SCY leave them vulnerable and susceptible to an array of health compromising conditions. • Forceful placements are psychologically and potentially physically harmful for SCY.
**Public Policy**	***Article 37 and 40 on juvenile justice:*** states should ensure the use of restorative rather than punitive juvenile justice, and should support protection rather than punishment of street-connected children and youth.	“We don’t get along well with the police because when they go down there, they just want to beat up someone... They go there and beat up people; there are even those who used to rape girls in town.” (Street-connected young woman 2)	• SCY are targeted with repressive street sweeps and are subject to police misconduct, which exposes them to physical violence, and leaves them with social and health inequities with life-long consequences. • Physical, psychological and sexual violence perpetrated by law enforcement has a lasting impact on the physical, sexual, and psychological health of SCY. • Criminal records may impact SCY’s life chances and have long-term consequences on their socioeconomic position thereby affecting their health.
**Public Policy / Socioeconomic Position**	***Article 2 on non-discrimination (25.) non-discrimination on the grounds of social origin, property, birth or other status:*** states must respect and ensure the rights of street-connected children and youth are upheld without discrimination of any kind.	“We might be seated here and when a police officer comes, he will see us as bad people and starts chasing us and beating us,yet we have not done any wrong. You go to prison for like 6 months, won’t you leave there as a bad person.” (FGD, street-connected young man)	• Discriminatory practices have life-long consequences on SCY’s socioeconomic position. • Discrimination leaves SCY without adequate access to social and health services which has a direct impact on their health and well-being.
**Public Policy / Socioeconomic Position**	***Article 2 on non-discrimination (26.) systemic discrimination:*** states are required to protect street-connected children and youth from direct and indirect forms of discrimination, including disproportionate policy approaches involving repressive efforts, including criminalization, street sweeps, and targeted violence.	“Two weeks ago, we rounded up street children. The community was concerned with the insecurity created by the street children. While street children do not commit all crimes, the situation overtime is problematic because of the numbers of children on the street. My job is to address issues of security. Community stakeholders told me that I should not arrest the street children as the police lack appropriate facilities, my priority is to protect the community.” (Police Officer)	• Repressive strategies to tackle homelessness may have a direct impact on SCY’s health when they are exposed to or experience violence as a result of round ups and targeted violence by enforcement officers. • Criminalization of street-involvement may have life-long lasting consequences on SCY’s socioeconomic position.
**Socioeconomic Position**	***Article 28 on education:*** states should make adequate provision, including support to parents, caregivers and families, to ensure that street-connected children and youth can stay in school and that their right to quality education is fully protected.	“Poverty at home, maybe they don’t have food, money to access education nor materials so the child will decide to go to the streets because he will feel better in the streets by begging from people. Also, maybe the parent did not give the child right to education and the child feels he has nothing to do at home, so they go to the streets to find something to do and earn a living.” (Religious Leader, Stakeholder)	• A lack of education impacts SCY’s long-term life circumstances and influences their ability to attain employment, and socioeconomic position; thereby impacting their health and ability to access resources to health. • SCY whom lack knowledge and skills attained through education may have reduced health knowledge, be ill equipped to navigate health services or communicate with health providers.

**Table 4:** Intermediary determinants of health, human rights, and their impact on SCY’s social and health inequities

Intermediary Determinants of Health			
Domain	Human Rights	Supporting Quotes	Impact on social and health inequities
**Material Circumstances**	***Article 27 on the right to an adequate standard of living*** ***(49.) Support to Parents, caregivers, and children:*** states should ensure that all children have a standard of living adequate for their physical, mental, spiritual and moral development.	“The basic needs, they don’t have food because most of the times you will find them eating from the bins. For clothes they have rags and they don’t have shoes. They also sleep outside. They don’t get loved due to separation so some of them are lonely; they don’t mingle with other people freely.” (Police Officer)	• A lack of essential basic needs, such as food, clothing, and shoes leave children and youth vulnerable to malnutrition, and exposed to health compromising conditions and at risk for acquiring infectious and non-infectious diseases. • SCY are also at risk of psychological consequences associated with street-involvement and a lack of an adequate standard of living.
**Material Circumstances**	***Article 27 on the right to an adequate standard of living (50.) Adequate Housing:*** states should sure that children and youth connected to the street have a right to live somewhere in security, peace and dignity.	“I live near them. I meet them in the morning while going to town; they can come and sleep in the vibandas (stalls) then go to town in the morning. When we walk at night, we warn them about sleeping there because someone being chased can also hide there.” (Community Leader 1)	• SCY lacking adequate housing and whom sleep in precarious or makeshift structures are at risk for numerous morbidities due to exposure to the elements and inadequate sanitation. • A lack of secure housing leaves SCY vulnerable to experiencing physical and sexual violence.
**Social-environmental or psychosocial circumstances**	***Article 6 on the right to life, survival and development (29.) on the right to life:*** states should ensure street-connected children and youth are free from acts and omissions intended or expected to cause their unnatural or premature deaths.	“Some of them are offenders. They did a mistake and ran away, so you have to sit with the family for several sessions, prepare them and tell them we have found your child any maybe tell us the history. ‘Ah that one is a thief, that one use to steal chicken, that one stole maize, even if he comes back’. Like they are some we took back to Baringo, and we didn’t know fully the felony they had committed. You know they were lynched!... Yeah the villagers in the community just tied them and lynched them.” (Children’s Officer)	• SCY experience unnecessary psychosocial stressors associated with infringement on civil and political rights, vigilante justice, and extrajudicial killings. • SCY disproportionately experience violence, which impacts their physical and mental health and often results in preventable and premature mortality.
**Social-environmental or psychosocial circumstances**	***Article 6 on the right to life, survival and development (32.) ensuring a life with dignity:*** states have an obligation to respect the dignity of street-connected children and youth, including in relation to procedural and practical funeral arrangements to ensure dignity and respect for children who die on the streets.	“My issue is with the morgue, when a street child dies, they are thrown inside a container and when we go to collect the body it becomes an issue because post-mortem has to be paid for and maybe what we have collected isn’t enough. We want it to be buried in a proper way. So, they will refuse to give us the body and eventually end up throwing it away. When we go to the HOD they tell us to look for its family and maybe they came, and they can’t afford to pay the charges. If they see some are smartly dressed, they say that we have to pay. Maybe the body has stayed for like 40 days and the charges have increased, they will even tell you that they are going to throw away that body. Sometimes we have to protest so that the body is released.” (Peer Navigator)	• SCY experience social inequities even in death due to income inequality and an inability to pay mortuary fees. • As a result, their peers experience unfair psychosocial stress to support their burial and a right to a dignified end of life.
**Social-environmental or psychosocial circumstances**	***Article 9 on separation from parents*:** states should not separate children from their families on the basis of their street-involvement, nor should states separate babies or children born to children themselves in street situations.	“For a mother with a child it also depends why that mother is there and from the experiences of interviewing them, these mothers use their babies to get sympathy from the public so whatever action we take will be a stern one like getting a court order to rescue those children and have the mother face the full face of the law. For the babies I take immediate actions because the environment is basically hostile to them.” (Children’s Officer)	• Separating street-connected babies and children from their parents or families is stressful for both their parent and the child and can have long-term psychological consequences for both.
**Social-environmental or psychosocial circumstances**	***Article 31 on rest play and leisure:*** street-connected children and youth have a right to utilize informal settings for play, and states should ensure they are not excluded in a discriminatory way from parks, and should adopt measures to assist them in developing their creativity and practising sport.	“Like last month we had a tournament, and some sponsored us and gave us playing kits and food. Our problem is not food alone; it should be something that makes sense and not just bread daily. You can give us food but also something to help us. Like supporting some of us who play football.” (FGD, Street-connected young men)	• Access to resources and ability to engage in rest, play, and leisure can reduce stressful life circumstances thereby ameliorating SCY’s health and well-being.
**Social-environmental or psychosocial circumstances**	***Article 19 and 39 on freedom from all forms of violence:*** states have the responsibility to protect street-connected children and youth from all forms of violence, including corporal punishment, familial violence, providing mechanisms for reporting violence, and holding perpetrators of violence accountable.	“Some are orphans, some come from dysfunctional families, maybe families where there is a lot of issues of abuse...Others you find they will tell you that there is a lot of violence at home. So, a child opts to run away and then eventually they end up in the streets.” (Children’s Officer)	• SCY may experience physical violence prior to street-involvement as well as once on the street due to their vulnerability and socioeconomic position. • Physical violence is linked to long-term physical and psychological health consequences including post-traumatic stress disorder.
**Social-environmental or psychosocial circumstances**	***Article 34–36 on sexual abuse, sexual exploitation, trafficking and other exploitation:*** states have the responsibility to protect children and youth from sexual violence, exploitation, and trafficking.	“If it’s a girl on the street, I become so irritated. She is very vulnerable. In Bungoma, there is a high rate of defilement of girls by the community at large, by family, by school children, and other teenagers.” (Police Officer)	• Experiencing physical and sexual violence is linked to long-term physical and mental health consequences. • SCY may experience sexual abuse and exploitation prior to street-involvement as well as once on the street due to their vulnerability and socioeconomic position.
**Social-environmental or psychosocial circumstances**	***Article 32 on child labour:*** states have responsibility to protect children and youth from economic exploitation and child labour.	“Others will move to other towns like if you go to Molo, Maunarok, mostly there is the issue of child labor. So, they will prefer to go to somewhere like Maunarok, where they know there are a lot of farms. And most of these people, they tend to use these children as casual laborers; you know they get something small.... if you go to Njoro, Molo where we have the flower farms, you will find many children, and the majority will tell you that we used to live on the streets.” (Children’s Officer)	• Child labour exposes SCY to stressful life circumstances including the possibility of violence or threats of violence. • Child labour may result in exposure to potentially harmful chemical, environmental and ergonomic factors and working conditions that are hazardous to their physical and psychological health.
**Behavioural and biological factors**	***Article 33 on drugs and substance abuse:*** street connected children and youth should have access to free healthcare services and states should increase the availability of prevention, treatment, and rehabilitation services for substance use.	“The things we use are very strong especially gum. It is stronger than alcohol and people who sniff gum are hard to talk to. They just do what they want. We use a lot of things, not just glue. Not all of them will understand things, you may tell me this and that but after sniffing gum I forget everything.” (FGD, street-connected young men)	• The health damaging effects of alcohol and substance use are well established. • SCY report that they use substances as coping and survival behaviour in response to the harsh environment on the streets. • These detrimental substance use practices are associated with their street-involvement and thereby socioeconomic position.

#### Socioeconomic and political context

##### Governance

Governance refers to “[the] system of values, policies, and institutions by which society manages economic, political, and social affairs through interactions within and among the state, civil society and private sector. It is the way a society organizes itself to make and implement decisions” [58]. CRC General Comment No. 21 (2017) Article 4 on appropriate measures outlines the responsibility of the State to provide appropriate legislative, administrative and other policies to ensure children have essential levels of each of the social, economic, and cultural rights. Participants interviewed recognized the responsibility of the State in intervening to protect SCY, as stated by one healthcare provider:

The government has to be involved because the moment the street kids are on the street we are creating a generation for criminals, those who won't get proper care, social and economic support and they end up becoming worse [off] than they were, if no proper interventions are made. (Clinical Officer)

Participants identified several limitations within the system of governance that act as barriers to realizing SCY’s rights. A government Children’s Officer recognized the State’s position of power to intervene. However, he also recognized the lack of political resolve for anyone to take responsibility for SCY:

It's a horrible situation because they live on the streets without the support of county or national government and they are a group of persons that have been rejected by the society... We in positions of authority and more so as a department having the powers of actually removing them from the streets provided there is political goodwill and the [will of] members of the society and the community at large. Because there seems to be no goodwill, the principal of every man for himself and God for us all, to the extent that nobody wants to take responsibility that these children are either in their docket or generally that they have the power to assist them. (Children’s Officer)

It was suggested that the issue of government inaction was not one of resource constraints, but one of power, where the State exercises power to shape the political agenda and power over decision-making regarding SCY. Only when the problem of child and youth street-involvement impacts an officials’ own position of power will they act, as explained by one Children’s Officer:

It's not even about the economics, it's our leaders' concern about these people and you will realize that they don't even talk about them. They only talk about them when they [officials] have been affected. (Children’s Officer)

Children’s Officers across counties agreed that the devolved system of governance has resulted in the issue of responsibility for SCY being contested, thereby leaving a gap in policy and services for this population. Officials concurred that cooperation between arms of government on this issue is required:

I think first of all as much as they say issues of street children are not devolved, that’s why you don’t see anyone speaking about it, at the department of children’s services, there is no place where you will hear them speak about these street children. They will tell you we don’t have that capacity, it’s not our work... I would propose it becomes a joint effort. They involve the national government and the county governments. (Children’s Officer)

As a result of political inaction and deficiencies in governance, SCY lack essential elements of social, economic and cultural rights.

##### Macroeconomic policies

The system of governance shapes and manages macroeconomic policies in Kenya and controls the economy. Participants point to Kenya’s economy and development level as a structural determinant of children and youths’ street-involvement:

It’s a problem because of our economy as a third world country; we are not able to get them out of the streets to better places. (Nurses)

Kenya has seen inconsistent economic growth over the past decade with a fluctuating gross domestic product; this has been in conjunction with a rising cost of living, particularly with respect to commodity prices [59, 60]. The unstable economy likely leaves families unable to meet their needs as a Clinical Officer suggests: “I can also say the cost of living is high; the parents are poor so they will go to the streets.” Moreover, participants recognized that it was the role of the State to improve the economy to reduce the burden on impoverished households as communicated by a community member:

I think they should be assisted, if the economy was better off I don’t think children would be on the streets, the economy should be good so that people can afford. They go to the streets because life became hard at home so the government should improve the economy. (General Community member)

##### Social policies and societal and cultural values

Kenya’s social welfare policies and programs such as the cash-transfer to orphaned and vulnerable children, may be missing many vulnerable households with children and youth at-risk of migrating to the streets as a result of poverty [61]. As one Children’s Officer suggests, the social welfare program needs to be expanded to meet the growing number of impoverished households requiring support:

Even if the parents have died, we still have extended families. The national government has programs like cash transfer programs whereby the government gives 2,000 shillings every month that is paid after every 2 months, I think it needs to enable us to target more families because the poverty level is getting high so we need to target more deserving families into the program. We also need programs centered on the street families. (Children’s Officer)

While the Children’s Officer also pointed to the extended family to care for orphaned children, traditional cultural and kinship values that previously acted as a social safety net for vulnerable children have eroded. Increasing economic pressures and individualistic values have shifted sociocultural norms resulting in children and youth turning to the streets as explained by a Clinical Officer:

The community as a whole has also failed because in the event that a child becomes an orphan or the family is not in a good position, they don’t come to solve the problem before it goes out of the boundary. Everybody lives for himself and God for us all, so what happens to the minors? They go to the streets and live their lives there. (Clinical Officers)

Once on the street, few social welfare programs exist to directly assist SCY. The lack of social welfare programs focused on SCY and impoverished families, combined with the dissolution of the traditional social-cultural safety net, leaves many vulnerable children and youth without a support system due to insufficient public policies.

##### Public policies

Numerous public policies result in systemic discrimination, human rights violations, and impact SCYs’ social and health inequities (Table 3). In text, we explore in-depth how public policies in relation to Article 20 on the right to special protection and assistance to children deprived of a family environment and Article 2 on non-discrimination contribute to inequities.

##### Article 20 on the right to special protection and assistance for children deprived of a family environment

When SCY are without parent(s)/guardian(s), the State is the de facto guardian and is obliged to ensure safe alternative care to a child temporarily or permanently deprived of his or her family environment; this does not include detention cells or closed centers where children and youth are deprived of liberty. Children’s Officers cite resource constraints in counties and a lack of appropriate shelters preventing them from ensuring safe alternative care for SCY:

You see now in [county redacted] we don’t have a rescue center, we don’t have a rehabilitation facility, so there is nothing much we can do, yet you are an officer in that capacity who is supposed to be protecting these children.

(Children’s Officer)

When rescue centers do exist, in many towns they aren’t State-run and may not have the capacity to care for all SCY as reported by a Children’s Officer:

The nearest is actually Machakos if you are talking about the national government, even Eldoret doesn't have a rescue center nor Nakuru and Kitale. What in those towns some people call rescue centers are owned by NGOs and sometimes private, but remember they are private entities and can only work to a certain level. So, what we want, and we are looking forward to, are street policies for street persons and establishment of rescue centers like you have asked me which are very necessary. (Children’s Officer)

In cases where no rescue center exists, SCY may end up in temporary holding cells, as a Police Officer describes:

We don’t have a children’s office in police stations in this country. Children need their own separate room, even with some beds. Sometimes they take three days to get help, so they need somewhere to sleep. If they are too small, we can use the hospital. We have a children’s cell, but that’s not the child protection unit. (Police Officer)

Insufficient or non-existent alternative care, or the use of children’s detention cells contravenes the right to special protection and assistance for children deprived of a family environment. Contrary to this obligation, the response to the issue of SCY is often characterized by criminalization, repressive and discriminatory policies and practices, which we will now explore.

##### Article 2 on non-discrimination

**Fig. 2:**
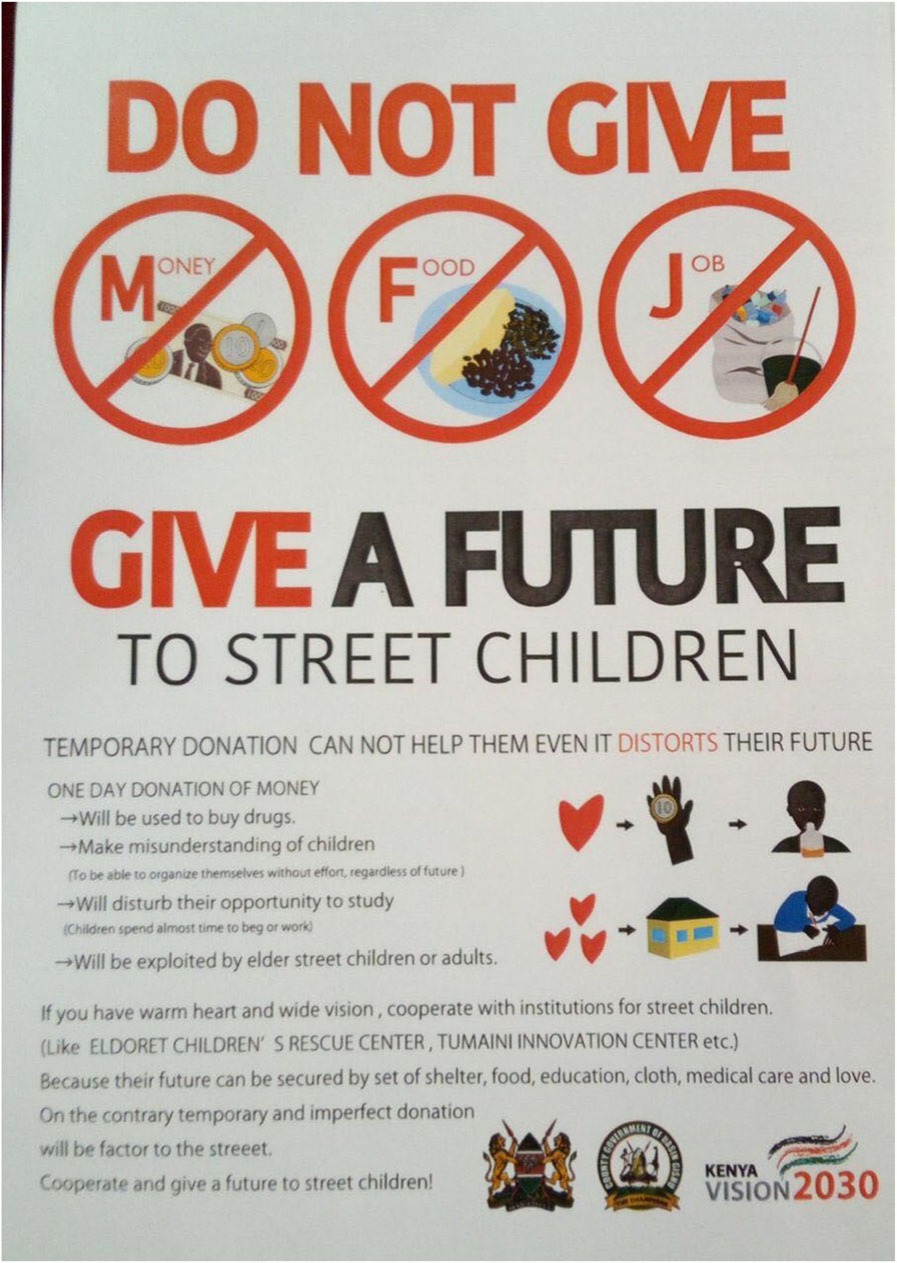
County policy document on SCY

States are required to respect and ensure that the rights of the child outlined in the CRC are upheld without discrimination. Yet, children and youth in Kenya are discriminated against on the basis of their street-involvement, and thereby ‘other status’ (Article 2.25). Figure 2. shows the repressive public policy in one county concerning SCY, which contravenes Article 2 (25) and (26) on non-discrimination. Moreover, this policy is contrary to the right to an adequate standard of living (Article 27), which includes the provision of food, and Article 6 (31) on the right to survival and development, and potentially infringes on Article 32 on child labor and the criminalization of begging.

Direct discrimination in the form of street sweeps, criminalization of street-involvement, and targeted violence by police are common across counties in Kenya as documented in the numerous newspaper articles analyzed [41–43, 45–48, 50]. Fears of public insecurity and a towns’ image are reasons typically cited for these repressive actions as explained in a popular daily newspaper in Kenya:

The reason given by the Mombasa county law enforcers for the arrest of more than 150 street families, most of them children, was “to fight insecurity, especially during this festive season”. (Daily Nation, December 31, 2015)

Discrimination against SCY in Kenya is far-reaching, and not limited to repressive strategies implemented by county governments. As a result of their street-involvement and social identities, SCY may continue to experience discrimination when they return to school and integrate into society, as a stakeholder explains:

If you go to school, children who saw you on the streets will always call you *chokoraa*. These are professionally trained teachers, but if you do something nasty, they will remind you that you were a street child. (Stakeholder)

The significance of being connected to the streets has long-term consequences on a young person’s life chances and social and health inequities. Once a child or youths’ social identity is defined as *chokoraa*, they are positioned in the social hierarchy as a social underclass.

#### Socioeconomic position

##### Social class, gender, and ethnicity

SCY in Kenya face multiple and intersecting forms of discrimination based on their ‘other status’ as *chokoraa*, and on the basis of social class, gender, and ethnicity. In general, SCY migrate to the streets as a result of poverty in impoverished households of a low social class [5, 6]. Once on the street, they are further subjugated to an even lower social class with extremely limited power, control, and prestige. SCY are generally perceived and characterized as juvenile delinquents and shunned by the public, as described by one street-connected young woman:

You know being a street child doesn’t mean that you are dirty; it depends on how you keep yourself. There are those who love water and others don’t. So, if we board a car, they won’t want us to sit at the front, they don’t see us as normal passengers. They see you as a thief and you smell so you can sit at the back. So, because I am with them, I will also sit at the back. If you go to the shop, they won’t attend to you fast because they think you don’t have money. The market women chase these children away when they go to beg. It’s like they are no one’s children. (FGD, Street-connected young women)

Their identity as *chokoraa* intersects with their gender. Discrimination on the basis of gender is prominent on the streets. Girls and young women connected to the streets, generally elicited feelings of ‘pity’ and ‘sympathy’ from participants because they are ‘weak’ and ‘victims’ who are subject to violence, rape, and have infants and children in their care. In contrast, boys and young men were characterized as ‘strong’, ‘dangerous’, and able to take care of themselves as explained by a Children’s Officer:

For a girl you will really sympathize, and you would even want to get immediate assistance for them because of even that cultural perception that is not the environment especially for a girl child. For the boys we kind of assume that they are tough, and they can sort of handle it for some time. (Children’s Officer)

These gender divisions result in differential treatment and disparate social, economic, and health outcomes for SCY, all rooted in socially constructed gender norms and roles. Discrimination on the streets also extends to ethnicity. Kenya has a complex and long-standing history of ethnic conflict and division on the basis of tribe rooted in historical colonialism [62]. Tribalism is used as a tool to abdicate county level responsibility for SCY as described by a stakeholder:

There is that perception that these children are not from this community, that they have come from other tribes, they are not ours so we cannot allow them to live here... The government says that they should go back to their people; they have come to make the town dirty, yet their children are safe at home. (Stakeholder)

This compounded discrimination on the basis of SCYs’ ‘other’ status, social class, gender, and ethnicity, is the result of socially produced differences constructed by the socioeconomic and political context. As a social underclass, SCY have limited access to education, opportunities for employment and income generation.

##### Education, occupation and income

Children and youth have a right to accessible, free, safe, relevant and quality education (Article 28). Attaining a formal education is an important component of an individuals’ socioeconomic position, influences life circumstances and has long-term impacts on a persons’ health. As a stakeholder explains, fees and the need to purchase school uniforms prohibit many parents from being able to send their children to school:

School opportunities, the parents are not able to afford a new pair of shorts, no shoes, no fees and all those factors will make the boy not hang in the right places. They don't go to school. (Stakeholder)

In turn, with little to no education SCY have few opportunities for employment, and when they do, it is usually informal exploitive labor that may expose them to harmful social-environmental circumstances as described by a community leader:

For the youths it's the unemployment on their side. We have been having the children on the streets for so long and some have been used by other people to do good or bad things, some work for them and are underpaid. (Community Leader)

The precarious informal work undertaken by SCY results in extremely low levels of income, as one former street-connected young man explains:

They depend on collecting plastics where a kilo goes for 10 shillings (~0.10 USD); a sack full of plastics can’t even get to 10 kilos, so per day they can make even 5 (~0.05 USD) shillings. (Former street-connected young man)

SCYs’ poor socioeconomic position, shaped and maintained by the socioeconomic-political context in Kenya, determines their differential exposure and susceptibility to intermediary health compromising factors.

#### Intermediary determinants of health

The intermediary determinants of health include material circumstances, social-environmental or psychosocial factors, and behaviours and biological factors that affect health (Table 4). We comprehensively explore Article 27 on the right to an adequate standard of living in association with material circumstances, and Article 6 (29) on the right to life, survival, and development in association with psychosocial factors in text.

##### Material circumstances

*Article 27 on the right to an adequate standard of living.*

SCY have a right to an adequate standard of living for their physical, mental, spiritual and moral development. This includes State support to parents, others, or directly to the child to ensure they have adequate nutrition, clothing, housing, free and accessible medical care, and education. Participants across counties unanimously expressed that SCY lack basic essential needs, which leave them exposed to health compromising conditions as explained by a Police Officer:

I feel for them, it is not right for them to be on the street. They are vulnerable children; they are supposed to be in school. They have many risk factors for disease; they have no food or shelter. They eat dirty food and so are exposed to diseases. (Police Officer)

At the foundation of material circumstances is the right to adequate housing. Housing has substantial impacts on health, and SCY have the right to live somewhere in security, peace and dignity. Typically, SCY are homeless or precariously housed in makeshift structures.

Like for me I come from California in Huruma where they dump wastes and many people sleep there, the place is dirty with many flies and they eat from there with pigs and dogs, so it is easy to get sick.... They survive without shelter; they have nowhere to put their belongings. (Former Street-connected young man)

SCYs’ right to an adequate standard of living through support to parents, caregivers, and children, and the right to adequate housing are unmet. These inadequate material circumstances create stressful living conditions and contribute to psychosocial stressors and poor physical condition.

##### Social, environmental and psychosocial factors

*Article 6 on the right to life, survival and development.*

SCY have the right to be free from acts and omissions intended or expected to cause their unnatural or premature death, and to enjoy a life with dignity. Participants reported that SCY succumb to mob and vigilante justice. A County official stated that he is unable to protect SCY from public revenge when they commit crimes:

You know in some situations you cannot help, if one of them kills or steals from the member of the public, the public will retaliate.

(County Children’s Coordinator)

Yet, SCY may be blamed for crimes they did not commit due to the public’s perception of them as thieves and juvenile delinquents as explained:

When bad things happen, they get blamed for it and they get mistreated. It is very easy for them to get killed. Some people steal in town, so the community assumes it’s the street children who do that, so many get killed for something they haven’t done.

(Former street-connected young woman)

Regardless of the responsibility for criminal activities, the use of mob and vigilante justice is present. SCY frequently succumb to death due to assault [24]. Moreover, the State may be complicit in some SCYs’ deaths, as reports of extrajudicial killing of SCY have been documented in the news [45]. SCY require the State’s protection from acts that cause their unnatural or premature death, and circumstances that infringe on their civil and political rights. The passivity of the State in vigilante justice, extrajudicial killings, or murder of SCY by adults or peers, contravenes Article 6 on the right to life, survival and development.

## Discussion

Our findings demonstrate the numerous structural, social, and intermediary determinants of health impacting SCY in Kenya. Utilizing the combined frameworks, the WHO conceptual framework on SDH and the CRC General Comment No. 21 (2017) on Children in Street Situations [2, 29], we have shed light on how the numerous social and health inequities experienced by SCY in Kenya are produced, maintained, and shaped by structural and social determinants of health and violations of their human rights. Principally, our findings suggest that the vast social and health disparities experienced by SCY in Kenya are a consequence of failures of governance and the State to take all appropriate legislative, administrative, and other measures for the implementation of the rights recognized in the CRC as a signatory State, and in the Kenyan Children’s Act [2, 30, 31, 63]. Our findings suggest abdication of responsibility, dysfunction in the system of devolved governance, and a lack of political will to exercise power and invest resources to intervene more appropriately. Repressive public policies instituted by the State include street sweeps, forced migration, criminalization of street-involvement, and targeted violence, and suggest these powers are exercised to uphold social order, political prestige and resources, resulting in oppression of SCY. The use of these and other repressive strategies, and the lack of special protection and assistance for children deprived of a family environment require an immediate response and remedial action from the State. Our findings are supported by a Save the Children report [64] which found that SCY lived in “sub-human circumstances and their very basic right to life is at risk every day” and that the government’s response has been to criminalize this marginalized group. The report also noted that the Kenyan government “failed to provide concrete and appropriate policies and strategies to uphold the rights of SCY, and initiatives to address the needs of SCY were “uncoordinated, human and financial resources were inadequate, and the rehabilitation of children was slow”. In other low- and middle-income countries, Human Rights Watch has documented several violations against SCY that contravene their human rights and ultimately impact their health and social well-being [13, 65–68]. Moreover, a report prepared by the Consortium on Street Children outlines several legislative and policy gaps and a failure to implement and uphold the CRC for SCY in low- and middle-income countries, which is in alignment with our findings [69].

Governments are responsible for protecting and enhancing health equity, and as a signatory of the CRC, Kenya has a legal obligation to implement policies to protect and provide assistance to SCY [2, 29–31, 63]. It is crucial for stakeholders and civil society, with the engagement and input from SCY, take collective action to advocate for a fundamental shift from repressive harmful responses to child-rights approaches as outlined in the CRC [2]. Upholding children’s rights and implementing child-rights policies and interventions to reduce social stratification, exposures, vulnerabilities, and unequal consequences of ill health will likely improve health equity for SCY [29].

Our findings show the issues impacting SCY’s health equity are far reaching, and beyond that of the health sector. Intersectoral government and civil society action on the structural and intermediary determinants is essential [29]. A commitment and collaboration between county and national level government with input from civil society and SCY to create contextually relevant and streamlined policies and programs using a child-rights approach is crucial. Structural determinants can be influenced and modified. Our findings suggest that action on key issues including poverty-reduction and social welfare programs, education, halting discriminatory practices, and ensuring special protection and assistance for children deprived of a family environment, should be immediately enhanced and strengthened. Expanding existing social policies, such as Kenya’s Cash-transfer to orphaned and vulnerable children program, to cover SCY and more vulnerable families should be a priority [61]. No less critical is action on the intermediary determinants. Material circumstances may have the most significant impact on marginalized populations, and policy to uphold the right to an adequate standard of living is a fundamental starting point [29]. Housing first initiatives have been successfully implemented with other homeless populations in high-income countries and similar policies and interventions may be adaptable to the context of SCY in Kenya [70]. Programs that aim to reduce/eliminate individual and systemic discrimination attached to being street involved should be developed to create an environment where SCY can be less stigmatized and public policy can legitimately recognize and uphold their rights.

Civil society may play a role in holding government and political leaders accountable for action on the SDH inequities [29]. The participation of civil society, SCY, and their families is a vital component of advancing policy to promote health equity, through stimulating political action. ﻿For example, the Children Act of Kenya encourages child participation in any procedure affecting a child [32] as their engagement is critical so that their views about their needs, access to resources and their human rights may be heard and taken into account. One possibility for stimulating political action may begin with researchers and stakeholders disseminating relevant and practical evidence to civil society organizations and policymakers, which can help put the issue of SCY on the political agenda and inform policy and decision-making. Moreover, civil society organizations engaged in legal and human rights work can petition policymakers to take action, while monitoring and evaluating their progress regarding the legal obligation of State as a CRC signatory [31, 71]. The process of empowerment and participation in shifting the political process likely presents challenges within the political context in Kenya and requires careful judgement and thoughtful engagement by social actors working to influence policy.

Avenues for intervening to advance health equity require context-specific action from the micro- to macro-level tackling both structural and intermediary determinants [29]. While this analysis sought to identify and understand how SCYs’ social and health inequities are produced and maintained by SDH, it did not identify specific avenues and strategies for action. Additional research to identify strategies for tackling the SDH inequities experienced by SCY in Kenya is required and will be fundamental in influencing policy and designing and implementing programs, to ameliorate the health and well-being of this vulnerable population.

This research has both strengths and limitations. This investigation included a wide range of social actors across western Kenya, which makes our findings contextually relevant to these counties. Our analysis was situated in the widely used and well-regarded WHO conceptual framework on SDH in conjunction with the CRC, making it appropriate and applicable to address health equity through legal and political changes. The use of newspaper media has both strengths and limitations. Newspapers included in this analysis provided supporting evidence of current events with respect to SCY and the sociopolitical policies targeting this population; however, it is important to recognize that news media may be biased in their reporting, and therefore this evidence should be interpreted with caution. Finally, our findings may not be representative or generalizable to all counties in Kenya as we only interviewed participants from five county capitals. The age group of SCY included in the study was also a limitation. SCY younger than 15 years of age may offer other perspectives, as evidence suggests that younger SCY were seen as “vulnerable” and may be worthy of assistance. Research that include evidence from and about younger age groups of SCY should be explored to provide a comprehensive understanding of health inequities and how/if they are addressed by the State.

## Conclusion

SCY in Kenya experience numerous social and health inequities that are socially produced, avoidable, and unjust. These social and health disparities arise as a result of structural and social determinants stemming from the socioeconomic and political context in Kenya that produces systemic discrimination and influences SCYs’ unequal socioeconomic position in society. In turn, SCY lack access to material resources, such as housing, basic material needs, and experience numerous psychosocial stressors, such as violence, which directly impact health equity. Remedial action to reverse human rights contraventions and to advance health equity through action on SDH for SCY in Kenya is crucial. This article contributes to understanding how social and health inequities experienced by SCY are produced and maintained and highlights how critical it is to uphold SCY’s human rights to improve their health equity.

## Supplementary information

**Additional file 1.** Interview Guide for Community Members Key Informant Interviews & Focus Group Discussions.

## Data Availability

The datasets used and/or analysed during the current study are available from the corresponding author on reasonable request.

## Abbreviations

WHO: World Health Organization
SCY: Street-connected children and youth
LMICs: Low- and middle-income countries
SDH: Structural and social determinants of health
CRC: Convention on the Rights of the Child
FGDs: Focus group discussions
AMPATH: Academic Model Providing Access to Healthcare
MTRH: Moi Teaching and Referral Hospital
