# Supplementary material for: Characterizing street-connected children and youths’ social and health inequities in Kenya: a qualitative study
Source: Int J Equity Health. 2020 Aug 28;19:147. doi: 10.1186/s12939-020-01255-8 (PMC7455900; doi:10.1186/s12939-020-01255-8)
Supplement: Supplementary file 1 — Additional file 1. Interview Guide for Community Members Key Informant Interviews & Focus Group Discussions. [file 12939_2020_1255_MOESM1_ESM.docx]

**Interview Guide for Community Members Key Informant Interviews & Focus Group Discussions**

***Perceptions***

**1.0** What do you think of when you hear the word street children? What comes to mind?

**1.1** What are your thoughts and feelings about children and youth who live in the streets?

**1.2** What are your thoughts on the reasons children and youth are living on the streets in Eldoret?

**1.3** How do you feel knowing that children and youth live on the streets in Kenya?

Is it a problem? What do you think it means for Kenya as a society?

***Experiences***

**2.0** Can you tell me about your experiences and interactions with street children and youth in Eldoret or other cities in Kenya?

**2.1** How often do you see and/or interact with street children?

**2.2** What do you feel when you encounter a street child?

**2.2.1** Follow up: Why do you think you feel that way?

**2.3** Do you feel differently if they are a girl or a boy? Young? Or teenager? Mother and child?

**2.4** How do you treat street children when you encounter them?

**2.4.1** Follow-up probe - Why do you treat them that way?

**2.5** Do you treat them differently based on their age or gender?

**2.5.1** Follow-up probe - Why do you treat them differently?

***Perceived Needs***

**3.0** What do you think should be done, if anything, about the street children situation in Eldoret or other cities in Kenya?

3.0.1 Follow-up as to why the respondent thinks X, Y or Z should be done.

**3.1** Do you feel there should be services and programs available to them? (ie. Drop-in centres, feeding programs, healthcare, education, rehabilitation centre)

3.1.1 Follow up: why or why not?

**3.2** If you were asked to devise a strategy and plan to assist street children what would you do?

**3.3** Who should be responsible for protecting children on the streets?

3.3.1 Follow up probe: What role should society play in protecting children on the streets? What role should government play? Street children themselves?

**3.4** Who should be responsible for designing and running programs and services?

**3.5** Do you feel that street children should be able to access healthcare services?

3.5.1 Follow-up with why or why not?

**3.6** Do you think that street children and youth should be treated like everyone else when going to care?

3.6.1 Follow-up - Why or why not?

**3.7** Do you think that street children require specialized healthcare programs, care, and services?

3.7.1 If yes, what kind? If no, why not? What programs do you think are important? What kinds of programs are feasible?

***For Healthcare providers only***

4.0 How do you feel when street children and youth come in for care?

4.1 What would help improve care for street-connected children and youth?

4.2 How do you feel when street children and youth come in for HIV testing and counseling?

4.3 What are your thoughts on providing HIV prevention to street-connected children and youth?

4.4 What do you think are their needs, if any, in relation to HIV prevention?

4.5 What kinds of programs do you think would be important for street-connected children and youth in relation to HIV prevention?

**Interview Guide for Key Informant Interviews and Focus Group Discussions with street-connected youth**

***Perceptions***

**1.0** What does it mean to be a street child?

**1.1** What are your thoughts and feelings about children and youth living on the streets?

***Experiences and Interactions with the community***

**2.0** I would like you to tell me about your experiences and interactions with residents (citizens, shopkeepers, government, healthcare providers) in town.

**2.1** Can you tell me more about how people treat you?

**2.2** Why do you think they treat you like this?

**2.3** Do different people treat you in different ways? (Ie. Do shopkeepers treat you differently than askaris?)

**2.4** How does their treatment make you feel?

**2.5** Do you feel that people care about you?

***Perceived Needs***

**3.0** What do you think should be done, if anything, about street children in Kenya?

**3.1** Who should be responsible for protecting/helping children on the streets?

**3.2** If you were asked to devise a strategy and plan to assist street children what would you do?

**3.3** Who should be responsible for designing and running programs and services?

***Access to Services & Healthcare Needs***

I am now going to ask you a series of questions related to accessing healthcare services and your healthcare needs.

**4.0** I would like you to tell me about your experiences accessing care at the hospital.

**4.0.1 Follow-up:** What makes you *uncomfortable* when coming for care? **/** What makes it difficult to access care? What makes (or would make) it easier to access care or what would make you more comfortable coming for care?

**4.1** What are your thoughts on how healthcare providers treat you?

I am now going to ask you some questions about your thoughts on HIV in your community.

**4.2** Do you think HIV is a problem in the street youth community? Why or Why not?

**4.3** We would like to hear about what types of HIV prevention activities would help you? What do you think you need to know more about?

What do you think would make a difference in your community?

What would you like to see more of? Less of?

- - How do you feel about community activities? (ie. football, music, drama activities with HIV prevention component?)
  - Peer education activities?
  - Life skills and financial literacy?

**4.4** Who do you think should be talking to street youth about HIV prevention?

**4.5** Do you feel more comfortable when your peers or someone closer to your age, talk to you about HIV prevention? Would you be willing to discuss HIV prevention and do activities with groups of peers your own age and gender?

**4.6** Would you be interested in participating in HIV prevention activities for street youth if they were occurring? Why or why not? What would make you more likely to participate in HIV prevention activities?
